# Practical Performance Analysis for Multiple Information Fusion Based Scalable Localization System Using Wireless Sensor Networks

**DOI:** 10.3390/s16091346

**Published:** 2016-08-23

**Authors:** Yubin Zhao, Xiaofan Li, Sha Zhang, Tianhui Meng, Yiwen Zhang

**Affiliations:** 1 Shenzhen Institutes of Advanced Technology, Chinese Academy of Sciences, Shenzhen 518055, China; zhaoyb@siat.ac.cn (Y.Z.); yw.zhang@siat.ac.cn (Y.Z.); 2 Shenzhen Institute of Radio Testing & Tech., Shenzhen 518000, China; zhangsha@srtc.org.cn; 3 Department of Mathematics and Computer Science, Freie Universität Berlin, Berlin 14195, Germany; tianhui.meng@fu-berlin.de

**Keywords:** indoor localization, Cramér–Rao lower bound, Bayesian estimation, non-line-of-sight, wireless sensor network

## Abstract

In practical localization system design, researchers need to consider several aspects to make the positioning efficiently and effectively, e.g., the available auxiliary information, sensing devices, equipment deployment and the environment. Then, these practical concerns turn out to be the technical problems, e.g., the sequential position state propagation, the target-anchor geometry effect, the Non-line-of-sight (NLOS) identification and the related prior information. It is necessary to construct an efficient framework that can exploit multiple available information and guide the system design. In this paper, we propose a scalable method to analyze system performance based on the Cramér–Rao lower bound (CRLB), which can fuse all of the information adaptively. Firstly, we use an abstract function to represent all of the wireless localization system model. Then, the unknown vector of the CRLB consists of two parts: the first part is the estimated vector, and the second part is the auxiliary vector, which helps improve the estimation accuracy. Accordingly, the Fisher information matrix is divided into two parts: the state matrix and the auxiliary matrix. Unlike the theoretical analysis, our CRLB can be a practical fundamental limit to denote the system that fuses multiple information in the complicated environment, e.g., recursive Bayesian estimation based on the hidden Markov model, the map matching method and the NLOS identification and mitigation methods. Thus, the theoretical results are approaching the real case more. In addition, our method is more adaptable than other CRLBs when considering more unknown important factors. We use the proposed method to analyze the wireless sensor network-based indoor localization system. The influence of the hybrid LOS/NLOS channels, the building layout information and the relative height differences between the target and anchors are analyzed. It is demonstrated that our method exploits all of the available information for the indoor localization systems and serves as an indicator for practical system evaluation.

## 1. Introduction

Locating a target using a wireless sensor network (WSN) is an efficient way to support multiple Internet of Things (IoT) applications, and many measurement and sensing techniques are proposed [1]. The techniques of measurement, such as angle-of-arrival (AOA), time-of-arrival (TOA), time-difference-of-arrival (TDOA) and received-signal-strength (RSS), play important roles in many applications, e.g., navigation, localization, target tracking and location-based service for mobile communication [2,3]. Just like other wireless applications, localization systems are sensitive to the signal noise. The major sources that influence the measurements result from the multi-path effect and non-line-of-sight (NLOS) during the propagation of the wireless signal due to the complicated infrastructures or environment. In addition, motivated by cloud computing and urban sensing technologies, multiple sensors are deployed widely, and much information in the complicated environment is also useful to calibrate the target position, e.g., map information and the trajectory records.

The main purpose of the localization system is the position estimation accuracy for a better location-based service. Thus, many research efforts focus on developing accurate location estimation algorithms [4]. The key idea is to fuse multiple pieces of information, derive the relationship to the target position and eliminate the error. The most popular tools are the nonlinear filters based on the recursive Bayesian estimation model, e.g., extended Kalman filters, particle filters and Gaussian filters, which fuse the prior information of the previous state and the current measurement data to derive positions [5,6,7]. One important factor that may influence the target position estimation is the NLOS signals. Thus, many algorithms try to identify the NLOS measurements and mitigate or adapt them based on the prior knowledge to improve the position estimation [8,9]. A hybrid method that combines the recursive Bayesian model and NLOS information is proposed according to the jump Markov model estimation [10]. Multiple information fusion methods are also studied, which fuse both TOA and RSS measurements to improve the estimation [11,12]. The map or building layout information is also modeled for the wireless localization system and is applied for estimation [13,14]. It can be found that with the development of the sensing technique, more and more information will be integrated into the sensors and used for localization in the future. Thus, the localization system requires a scalable architecture to fuse such information. Although the indoor localization system is designed via employing multiple pieces of information, a theoretical analysis for such heterogeneous information is not provided yet.

Before the practical implementation of the WSN localization system, theoretical analysis is also required to evaluate the performance of the heterogeneous information fusion. The Cramér–Rao lower bound (CRLB) as the optimal performance indicator for the unbiased estimator is widely applied in localization and positioning systems. Theoretical investigations have been researched as the nonlinear problem for wireless localization systems. Tichavsky et al. provided the formulation of recursive posterior CRLB for nonlinear filters based on the Bayesian framework [15]. Zuo et al. proposed a conditional CRLB, which considered that the posterior probability is conditioned on the prior probability [16]. For range-based wireless localization system, many research works have provided CRLB results for different scenarios. A generalized CRLB (G-CRLB) of the wireless system is proposed for the NLOS environment [17]. The hybrid LOS/NLOS environment is analyzed, and the authors indicated that with prior knowledge of the wireless transmission channel, the estimation performance can be improved [17]. Shen et al. defined an equivalent CRLB (E-CRLB) for a general framework of the wideband wireless network [18]. The multi-path and NLOS effect are both considered, and the CRLB with or without prior information is compared to the E-CRLB [18]. A linear CRLB (L-CRLB) is proposed, which considers the linearized effect and provided the lower bound for such an estimator [19]. Other similar works also give CRLB for different ranging techniques [20,21]. Although some other methods can be used for performance analysis [22], CRLB is still popular for wireless localization researchers due to its simplicity and general expression. Although the above-mentioned CRLBs try to provide the general fundamental limits of the localization systems, they assume only parts of the factors in the localization problem, which limit their applications. In this case, these CRLBs still cannot be analyzed precisely since more information makes the environment complicated, and they are influenced by many unknown factors. Therefore, a general scalable framework of CRLB is required to further collect more information and evaluate the practical environment.

In this paper, we propose a general analysis method to evaluate the practical WSN localization system, which can fuse multiple pieces of information. The first major contribution is that we construct a scalable framework to model the multiple information fusion in the localization problem generally. We derive the Fisher information matrix (FIM) based on the proposed abstract function of all of the wireless localization system model instead of using a specific wireless propagation model. Furthermore, we employ an extendable estimated random vector θ, which contains all of the unknown parameters, e.g., the previous estimated state, the current state and the unknown indeterministic parameters that may influence the estimation accuracy. Then, we divide θ into two parts: the estimated state vector, which indicates the final position estimation, and the auxiliary vector, which helps improve the estimation accuracy. When θ only contains the state vectors or together with NLOS indicators, the our method is equivalent to the above-mentioned CRLBs. However, when a large number of parameters is involved in the estimation, e.g., the map information, the previous state, the relative height difference or the prior information of the unknown random parameters, our method can still provide practical optimal performance of the localization systems. The major advantage is that it is suitable for complicated and dynamic environment and fully considers the prior information, hybrid unknown factors and the recursive feature of the tracking algorithms.

The second contribution is that we employ the proposed CRLB framework to analyze the TOA range-based indoor localization system as a case study. The simulation environment considers all of the possible factors, e.g., the target-anchor geometry effect, the building layout, the relative height differences between the target and anchors, the NLOS transmission channel, the related prior information and the recursive feature of the tracking algorithm. The impact of each factor for the estimation accuracy is illustrated in the simulation. We use the spatial position error distribution (SPED) as a metric to evaluate the performance. The SPED indicates not only the target-anchor geometry effect, but also illustrates the impacts of height differences between the target and anchors, NLOS transmission and building layout information for the indoor localization. In addition, the impacts of the related prior information are also evaluated via SPED. To indicate which factor is important to the location estimation, we numerically evaluate the NLOS ranging, the height difference and the prior information in multiple scenarios. For the dynamic moving trajectories, the recursive form of the CRLB is applied. Finally, the estimation improvement using multiple anchors is also presented in the simulation. The results indicate that the NLOS ranging measurement mainly influences the estimation accuracy, and the prior information of the NLOS channel and the target position play important roles for improving the estimation. The relative height differences also degrade the estimation accuracy if we ignore them. However, with reliable prior information, we can make the estimation accuracy approach the location performance without any relative height differences. In general, our proposed method is suitable to exploit all of the available information to analyze the performance of the WSN localization system.

The rest parts of this paper is organized as follows: Section 2 provides the WSN localization system model; Section 3 introduces the scalable CRLB framework; then, we use the CRLB to derive the formulation of a practical TOA system in Section 4; the simulation evaluations and analysis are illustrated in Section 5; and Section 6 concludes the whole paper. To make the content more clear, we list all of the fundamental notations in Table 1 for the mathematical formulation.

## 2. System Model

In the WSN localization system, the mobile device with an unknown position is called the target, such as a mobile sensor node or robot. The position state of the target is denoted by $x_{t}={\left[p_{t}^{X}\;p_{t}^{Y}\right]}^{T}$, where $p_{t}^{X}$ and $p_{t}^{Y}$ are the coordinates in the two-dimensional positioning system, and *T* is the transpose operator. The wireless sensor devices with known positions, which measure the ranges (or distances) to the target, are called anchors. For each anchor, the position is denoted by $a_{j}={\left[a_{j}^{X}\;a_{j}^{Y}\right]}^{T}$, where $a_{j}^{X}$ and $a_{j}^{Y}$ are the coordinates. In this paper, we assume the WSN localization system uses the time-of-arrival (TOA) method to measure the distance between the target and anchor. Thus, we will have the relative factors that may influence the TOA and the final location estimation.

### 2.1. Time-Of-Arrival Ranging

In the TOA measurement method, the distance between the target and anchor is calculated according to the wireless propagation time. Consider a synchronous wireless communication, where clocks at the target and anchors are strictly synchronized. Anchors send the ranging measurements periodically via a time-division multiplexing (TDM) method based on the related WSN protocol. The target receives a radio signal transmitted from one anchor via a single propagation path. Let $\tau_{t}^{j}$ be the time delay of the received signal from anchor *j* at time *t*:

(1) $$\tau_{t}^{j}=\frac{1}{c}\left[\left|\right|x_{t}-a_{j}\left|\right|+l_{t}^{j}\right]$$

where $c=3\times 10^{8}\;m/s$ is the propagation speed of the signal, $a_{j}={\left[a_{j}^{X}\;a_{j}^{Y}\right]}^{T}$ is the anchor position and ||·|| denotes the distance between two positions; $l_{t}^{j}\geq 0$ is the range drift, which is caused by the NLOS effect. The indicator $l_{t}^{j}=0$ for the LOS propagation, whereas $l_{t}^{j}>0$ for the NLOS propagation. For many indoor systems, the TOA ranging measurement is obtained through the packet transmission time based on the network protocol; thus, the TOA is denoted as the time observation instead of the received waveform, as depicted in [17,18]:

(2) $$z_{t}^{j}=c\tau_{t}^{j}+v_{t}^{j}$$

where $z_{t}^{j}$ is the range measurement for anchor *j*, the measurement function $h_{t}^{j}\left(\left(d\left(x_{t},k\right),l_{t}^{j}\right)\right)=c\tau_{t}^{j}$ and $v_{t}^{j}$ is the measurement noise for anchor *j*. The measurement noise $v_{t}^{j}$ follows the zero-mean Gaussian distribution $v_{t}^{j}∼N\left(0,R_{j}\right)$, where $R_{j}$ is the variance of the ranging noise.

### 2.2. TOA Noise

The localization performance mainly relies on the measurement noise. Thus, it is necessary to list the factors that are related to the TOA noise. TOA relies on the quality of detecting the direct path (DP) signal. Thus, the TOA ranging not only tolerates the attenuation of the signal strength, but also depends on the peak of all received multipath signals related to the bandwidth. Thus, the radio interference, the multipath effect and the scenario variability affect the ranging error. In addition, the TOA ranging noise also depends on the system noise. The system noise comes from the unsynchronized signals and the disordered received signals, which are transmitted from different sensors simultaneously. Apart from the NLOS effect, the comprehensive impacts on the ranging noise can be generally modeled as the normal distribution [23].

### 2.3. Relative Height

Another important factor that should be considered in the real-world system is that the localization happens in the 3D world. In many theoretical research works, it is assumed that the anchors and targets are on the same plane in many real localization applications. The goal is to calculate 2D positions, the X−Y coordinates of the target, which is already implemented in our daily apps. In this case, the height difference between anchor and target is ignored. However, the existing height difference in the real world actually affects the accuracy in the 2D position estimation. Here, we define the height difference between the anchor and target as relative height, which is a positive variable in the *Z* axis. Then, we use $k_{t}\geq 0$ to denote the coordinate of the target in the *Z* axis and $a_{j}^{Z}$ to denote the coordinate of the anchor. Thus, the relative height is $k_{t}-a_{j}^{z}$ at time *t*. If the relative height is zero or assumed to be zero in the simulation, we define the range measurement as 2D-ranging. The distance of 2D-ranging for each anchor is formulated as:

(3) $$d^{j}\left(x_{t},k\right)=\sqrt{{\left(p_{t}^{X}-a_{j}^{X}\right)}^{2}+{\left(p_{t}^{Y}-a_{j}^{Y}\right)}^{2}}$$

where $d^{j}\left(x_{t},k_{t}\right)$ denotes the distance function from anchor *j* to the target. If the relative height between the anchor and target is not zero, which is always applicable in the real case, the measurement depends on 3D coordinates, then we define the range measurement as 3D-ranging. The 3D-ranging for each anchor is formulated as:

(4) $$d^{j}\left(x_{t},k_{t}\right)=\sqrt{{\left(p_{t}^{X}-a_{j}^{X}\right)}^{2}+{\left(p_{t}^{Y}-a_{j}^{Y}\right)}^{2}+{\left(k_{t}-a_{j}^{Z}\right)}^{2}}$$

**Figure 1:**
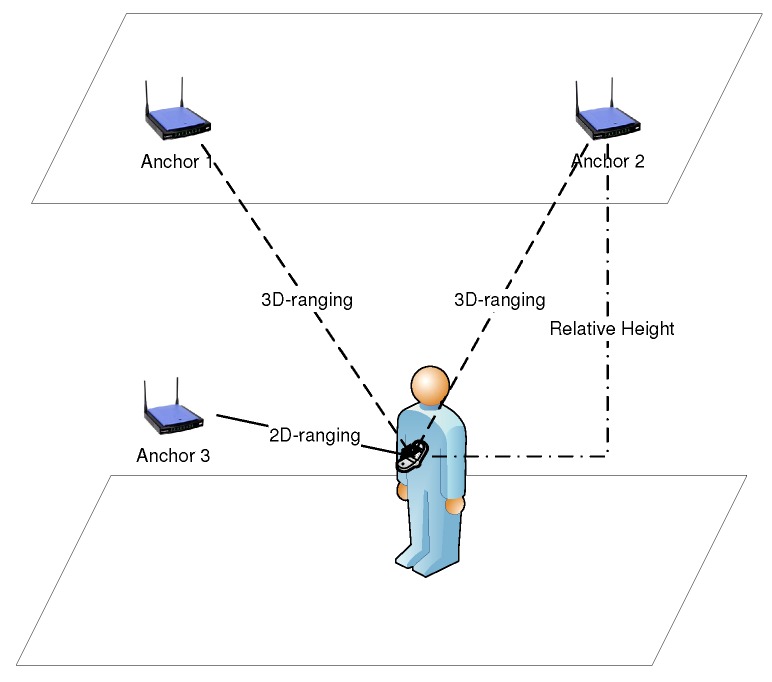
Demonstration of 3D-ranging.

Figure 1 illustrates the difference between 2D-ranging and 3D-ranging. Suppose a person or a robot is carrying a mobile device and walking in the building. Anchor 1 and Anchor 2 are deployed on the roof, and the distance to the target depends on the target position on the ground and the relative height. Even if the target is just below the anchor, the measurement is still not zero. Anchor 3 is deployed on the same plane of the target. In this case, the range measurement is 2D-ranging. However, 2D-ranging is an ideal case, and the anchors cannot be always on the same plane of the targets. No matter whether 2D-ranging or 3D-ranging, the position estimation is still for 2D in the playing field.

## 3. Scalable Framework of FIM

We attempt to construct the scalable analytical framework according to the Bayesian estimation process. The Bayesian estimation is an efficient way to fuse multiple pieces of information. Firstly, we build the predict-update model of the Bayesian estimation framework. Then, we derive the scalable Fisher information matrix.

### 3.1. Bayesian Model

According to the Bayesian estimation framework, the relationship between the estimated state $x_{t}$ and the measurement $z_{t}$ follows:

(5) $$x_{t}=f_{t}\left(x_{t-1}\right)+q_{t}$$

where (5) is the prediction function. In (5), the target’s movement is based on the transition function $f_{t}\left(\right)$, and $q_{t}$ is the prediction noise, which follows the normal distribution $N(0,Q_{t})$, where $Q_{t}$ indicates the covariance matrix.

In order to generally denote the wireless localization model, we propose an abstract measurement function of the unknown vectors:

(6) $$z_{t}=h_{t}\left(d\left(x_{t},k\right),\;l\right)+v_{t}$$

where (6) is the abstract measurement function, which is a general expression based on several range measurement techniques. In (6), $z_{t}={\left[z_{t}^{1}\ldots z_{t}^{j}\ldots z_{t}^{N}\right]}^{T}$ is the measurement vector, and *N* denotes the number of anchors. Note that $z_{t}$ can represent the RSS vector or TOA vector, and (6) can be rewritten according to the different measurement techniques. Then, $h_{t}\left(\right)={\left[h_{t}^{1}\left(\right)\ldots h_{t}^{j}\left(\right)\ldots h_{t}^{N}\left(\right)\right]}^{T}$ is the nonlinear observation function, which relates to the actual received signal waveforms at the target from the anchors; $v_{t}={\left[v_{t}^{1}\ldots v_{t}^{j}\ldots v_{t}^{N}\right]}^{T}$ is the ranging noise, which is assumed as independent noise; $d\left(\right)={\left[d_{1}\left(\right)\ldots d_{j}\left(\right)\ldots d_{N}\left(\right)\right]}^{T}$ represents the distance vector between the target and anchors. The auxiliary parameters are defined as the optional unknown factors that may influence the estimation. In the position estimation, the auxiliary parameters are not necessarily calculated. However, the information of such parameters can improve the estimation accuracy. We define two kinds of auxiliary parameters that may influence $z_{t}$ in (6). The first one is the nonlinear auxiliary vector $k={\left[k_{1}\ldots k_{j}\ldots k_{N_{k}}\right]}^{T}$, which affects the actual distance together with the position state $x_{t}$, and $N_{k}$ denotes the number of elements of k. The second one is the linear auxiliary vector $l={\left[l_{1}\ldots l_{j}\ldots l_{N_{l}}\right]}^{T}$, which affects the observed measurement $z_{t}$ and is independent of $x_{t}$, where $N_{l}\leq N$ indicates the number of the parameters. Since (6) is a general expression for several range measurement techniques, it should be specified and rewritten in a particular system. In this paper, (6) is simplified as the TOA formulation, which is discussed in the following sections. According to the Bayesian theorem, the posterior probability of $x_{t}$ is expressed as $p\left(x_{t}|z_{t},x_{t-1}\right)=p\left(x_{t}|x_{t-1}\right)p\left(z_{t}|x_{t}\right)$, where t−1 indicates the previous time interval, and $p(x_{t}|x_{t-1})$ is the prior probability [24].

### 3.2. Recursive FIM

Our analysis fully considers all of the possible unknown random factors that may influence the position estimation; hence, the parameter vector includes: the current state $x_{t}$, the previous state $x_{t-1}$ and auxiliary parameter vectors k and l. Thus, θ is expressed as:

(7) $$\theta ≜{\left[x_{t}^{T}\;x_{t-1}^{T}\;k^{T}\;l^{T}\right]}^{T}$$

The CRLB, which is given by the inverse of the Fisher information matrix (FIM), sets the lower limit for the variance (or covariance matrix) of any unbiased estimators of an unknown parameter (or unknown parameters) [25]. If $p(\theta ,z_{t})$ denotes the joint probability density function (PDF) of observations $z_{t}$ and the state θ, then the score function is defined as the gradient of its log-likelihood:

(8) $$U\left(x_{t}\right)≜\nabla_{\theta }\ln p\left(\theta ,z_{t}\right)$$

where $\nabla_{\theta }={\left[\frac{\partial }{\partial {`}_{1}},\ldots ,\frac{\partial }{\partial {`}_{N}}\right]}^{T}$ is the operator of first order partial derivatives. The FIM, J(θ), is the covariance of the score function:

(9) $$J\left(\theta \right)≜E\left\{\nabla_{\theta }\ln p\left(\theta ,z_{t}\right){\left[\nabla_{\theta }\ln p\left(\theta ,z_{t}\right)\right]}^{T}\right\}$$

where $E\left\{\cdot \right\}$ indicates the expectation operator. Additionally, the CRLB is just the inverse of FIM, and the estimation covariance cannot be lower than it:

(10) $${\mathrm{Cov}}_{\theta }\left(\tilde{\theta }\right)⪰{\left\{J\left(\theta \right)\right\}}^{-1}$$

where “A⪰B” should be interpreted as matrix A−B is non-negative definite.

Since $p\left(\theta ,z_{t}\right)=p\left(z_{t}|\theta \right)p\left(\theta \right)$ is based on the Bayesian theorem, it is easily seen that J(θ) can be decomposed into two parts:

(11) $$J\left(\theta \right)=J_{D}\left(\theta \right)+J_{P}\left(\theta \right)$$

where $J_{D}\left(\theta \right)$ represents the information obtained from measurement data and $J_{P}\left(\theta \right)$ represents the prior information.

Firstly, we use the notations $h=h_{t}\left(d\left(x_{t},k\right),l\right)$, $h_{j}=h_{t}^{j}\left(d\left(x_{t},k\right),l\right)$ and decompose $J_{D}$ using the chain rule as:

(12) $$J_{D}\left(\theta \right)=H\cdot J_{h}\cdot H^{T}$$

where $H=[\nabla_{\theta }h]$ and $J_{h}$ is the FIM conditioned on h:

(13) $$J_{h}=E\left\{\nabla_{h}\ln p\left(z_{t}|\theta \right){\left[\nabla_{h}\ln p\left(z_{t}|\theta \right)\right]}^{T}\right\}$$

The matrix H is further decomposed into four components:

(14) $$H={\left[H_{t}\;H_{t-1}\;K\;L\right]}^{T}$$

where $H_{t}={\left[\nabla_{x_{t}}h\right]}_{2\times N}$, $H_{t-1}={\left[\nabla_{x_{t-1}}h\right]}_{2\times N}$, $K={\left[\nabla_{k}h\right]}_{N_{k}\times N}$ and $L={\left[\nabla_{l}h\right]}_{N_{l}\times N}$. Since d is independent of the previous state $x_{t-1}$, $H_{t-1}=0$. Then, H is formulated as:

(15) $$H={\left[H_{t}\;0\;K\;L\right]}^{T}$$

For $J_{h}$, we can use diagonal matrices of order *N* to represent it:

(16) $$J_{h}=\Lambda =\mathrm{diag}\left(\lambda_{1},\ldots ,\lambda_{j},\ldots ,\lambda_{N}\right)$$

where the diagonal term $\lambda_{j}$ depends on $h_{t}^{j}\left(\right)$ and will be derived based on the typical system later. Then, $J_{D}$, which is a $\left(4+N_{k}+N_{l}\right)\times \left(4+N_{k}+N_{l}\right)$ matrix, is written as:

(17) $$\begin{array}{cc} J_{D} & =H\cdot \Lambda \cdot H^{T}\\ & =\left[\begin{array}{cccc} D_{11} & 0 & D_{13} & D_{14}\\ 0 & 0 & 0 & 0\\ D_{13}^{T} & 0 & D_{33} & D_{34}\\ D_{14}^{T} & 0 & D_{34}^{T} & D_{44} \end{array}\right] \end{array}$$

where:

(18) $$\left.\begin{array}{c} D_{11}=H_{t}\Lambda H_{t}^{T}\\ D_{13}=H_{t}\Lambda K^{T}\\ D_{14}=H_{t}\Lambda L^{T}\\ D_{33}=K\Lambda K^{T}\\ D_{34}=K\Lambda L^{T}\\ D_{44}=L\Lambda L^{T} \end{array}\right.$$

Without prior information, $J_{D}$ cannot indicate the CRLB directly, because $\det(J_{D})=0$ and $J_{D}$ is not reversible. However, the CRLB can also be attained through a calculation rule. The calculation rule is illustrated in the next subsection. Next, we derive $J_{P}$. The prior probability for θ is extended as $p\left(\theta \right)=p\left(x_{t}|x_{t-1}\right)p\left(k\right)p\left(l\right)$, then the prior information is written as:

(19) $$\ln p\left(\theta \right)=\left[\ln p(x_{t}|x_{t-1})\right]+\ln p\left(k\right)+\ln p\left(l\right)$$

where p(k) and p(l) are the independent prior information of $x_{t}$ and $x_{t-1}$. If we decompose θ into two sub-vectors: the state vector ${\left[x_{t}\;x_{t-1}\right]}^{T}$ and the auxiliary vector ${\left[k\;l\right]}^{T}$. Then, $J_{P}$ can be formulated as:

(20) $$\begin{array}{cc} J_{P} & =E\left\{\nabla_{\theta }\ln p\left(\theta \right){\left[\nabla_{\theta }\ln p\left(\theta \right)\right]}^{T}\right\}\\ & =\left[\begin{array}{cc} J_{P_{11}} & J_{P_{12}}\\ J_{P_{12}}^{T} & J_{P_{22}} \end{array}\right] \end{array}$$

where $J_{P_{11}}$ is the recursive form of $x_{t}$ and $x_{t-1}$, which is formulated by Tichavsky et al. [15]:

(21) $$\begin{array}{cc} J_{P_{11}} & =\left[\begin{array}{cc} M_{11} & M_{12}\\ M_{12}^{T} & M_{22}+J\left(x_{t-1}\right) \end{array}\right] \end{array}$$

where:

(22) $$\left.\begin{array}{c} M_{11}=Q_{t}^{-1}\\ M_{12}=\nabla_{x_{t-1}}f_{t}\left(x_{t-1}\right)Q_{t}^{-1}\\ M_{22}=\nabla_{x_{t-1}}f_{t}\left(x_{t-1}\right)Q_{t}^{-1}{\left[\nabla_{x_{t-1}}f_{t}\left(x_{t-1}\right)\right]}^{T} \end{array}\right.$$

where $J(x_{t-1})$ is the previous FIM of $x_{t-1}$. Additionally, $J_{P_{12}}$ are 0 matrices, since p(k) and p(l) are independent of $x_{t}$ and $x_{t-1}$:

(23) $$\begin{array}{cc} J_{P_{12}} & =\left[\begin{array}{cc} 0 & 0\\ 0 & 0 \end{array}\right] \end{array}$$

The prior distribution $p(x_{t}|x_{t-1})$ is also independent of l and k; thus, $J_{P_{21}}=J_{P_{12}}^{T}=0$. Finally, the last element $J_{P_{22}}$ is expressed as:

(24) $$\begin{array}{cc} J_{P_{22}} & =\left[\begin{array}{cc} J_{K} & 0\\ 0 & J_{L} \end{array}\right] \end{array}$$

where $J_{K}$ and $J_{L}$ are the FIMs conditioned on k and l, respectively:

(25) $$\left.\begin{array}{c} J_{K}=E\left\{\nabla_{k}\ln p\left(k\right){\left[\nabla_{k}\ln p\left(k\right)\right]}^{T}\right\}\\ J_{L}=E\left\{\nabla_{l}\ln p\left(l\right){\left[\nabla_{l}\ln p\left(l\right)\right]}^{T}\right\} \end{array}\right.$$

The formulations of $J_{K}$ and $J_{L}$ are based on the features of k and l, which are discussed in the later sections. Then, substitute (17) and (20) into (11); we obtain:

(26) $$J\left(\theta \right)=\left[\begin{array}{cccc} M_{11}+D_{11} & M_{12} & D_{13} & D_{14}\\ M_{12}^{T} & M_{22}+J\left(x_{t-1}\right) & 0 & 0\\ D_{13}^{T} & 0 & D_{33}+J_{K} & D_{34}\\ D_{14}^{T} & 0 & D_{34}^{T} & D_{44}+J_{L} \end{array}\right]$$

However, J(θ) is a matrix of high dimensions, while only a small submatrix $J^{-1}\left(x_{t}\right)≜{\left[J^{-1}\left(\theta \right)\right]}_{2\times 2}$ is of interest. Using the form of the Schur complement of the submatrix [26], we decompose the FIM into two parts: the state matrix $J_{S}$ and the auxiliary matrix $J_{A}$:

(27) $$J\left(x_{t}\right)=J_{S}-J_{A}$$

where:

(28) $$\left.\begin{array}{c} J_{S}=M_{11}+D_{11}-M_{12}{\left(M_{22}+J\left(x_{t-1}\right)\right)}^{-1}M_{12}^{T}\\ J_{A}=\left[D_{13}\;D_{14}\right]{\left[\begin{array}{cc} D_{33}+J_{K} & D_{34}\\ D_{34}^{T} & D_{44}+J_{L} \end{array}\right]}^{-1}{\left[D_{13}\;D_{14}\right]}^{T} \end{array}\right.$$

Additionally, the formulation of each element can be found in (18), (22) and (25).

### 3.3. Calculation Rule

Equation (27) only holds when all of the elements in θ are to be estimated and the prior information for the whole θ is available. If the CRLB is used to analyze a specific localization system, not all of the elements are necessary for θ, and some vectors are absent sometimes. For instance, for the non-recursive scenario, the system does not consider $x_{t-1}$. In addition, when the system has a deterministic value of the auxiliary vectors, k and l are not estimated and useless for $J(x_{t})$. Thus, the calculation principle for our CRLB is that: when any vector in θ is absent, the related matrix in (27) turns into 0, and we will treat such a 0 matrix as the empty matrix, then we mitigate the empty matrix for further calculation.

Here, we consider two examples. The first example is that the estimated vector θ does not include $x_{t-1}$ and k, then $M_{12}$, $M_{22}$, $J(x_{t-1})$, $J_{K}$, $D_{13}$, $D_{33}$ and $D_{34}$ are 0 and mitigated from (27):

(29) $$J\left(x_{t}\right)=M_{11}+D_{11}-D_{14}{\left[D_{44}+J_{L}\right]}^{-1}D_{14}^{T}$$

which has the same form as the formulation of E-CRLB in the wideband NLOS environment [18] and L-CRLB for the linear formulation [19].

The second example is that the estimated vector θ does not include k and l, and $J_{A}$ is empty, then only $J_{S}$ remains, which is the form for the recursive nonlinear filter [15]:

(30) $$J\left(x_{t}\right)=M_{11}+D_{11}-M_{12}{\left[M_{22}+J\left(x_{t-1}\right)\right]}^{-1}M_{12}^{T}$$

## 4. Application to a TOA WSN Localization System

In this section, we apply the proposed CRLB to analyze the time-of-arrival (TOA)-based WSN localization systems. Note that this paper provides a scalable framework for theoretical analysis rather than a particular analysis. Thus, our goal is not to find the major impacts or factors via mathematical derivation, but through the numerical simulations. In addition, according to the formulation of (27), many factors are involved in the calculation. It is not easy to analyze them via a single formulation. Thus, after enumerating the important factors in the TOA system, we will evaluate the performance via numerical analytical simulations. The impacts of the practical conditions, e.g., the real 3D anchor deployment and the NLOS transmission based on the building layout, are formulated. The related parts in (26) are derived for both cases with and without prior knowledge.

### 4.1. Relative Height

As mentioned before, no matter whether 2D-ranging or 3D-ranging, the position estimation is still for 2D in the playing field, which means we just want to obtain $x_{t}={\left[p_{t}^{X}\;p_{t}^{Y}\right]}^{T}$ and not $k_{t}$. However, for CRLB analysis, we still consider if $k_{t}$ is involved in the calculation and check whether the estimation accuracy is influenced. Then, $H_{t}$ is obtained:

(31) $$H_{t}=\left[\begin{array}{ccccc} \frac{\partial d_{t}^{1}\left(x_{t},k_{t}\right)}{\partial p_{t}^{X}} & \ldots & \frac{\partial d_{t}^{j}\left(x_{t},k_{t}\right)}{\partial p_{t}^{X}} & \ldots & \frac{\partial d_{t}^{N}\left(x_{t},k_{t}\right)}{\partial p_{t}^{X}}\\ \frac{\partial d_{t}^{1}\left(x_{t},k_{t}\right)}{\partial p_{t}^{Y}} & \ldots & \frac{\partial d_{t}^{j}\left(x_{t},k_{t}\right)}{\partial p_{t}^{Y}} & \ldots & \frac{\partial d_{t}^{N}\left(x_{t},k_{t}\right)}{\partial p_{t}^{Y}} \end{array}\right]$$

where:

(32) $$\left\{\begin{array}{c} \frac{\partial d_{t}^{j}\left(x_{t},k_{t}\right)}{\partial p_{t}^{X}}=\frac{p_{t}^{X}-a_{j}^{X}}{\sqrt{{\left(p_{t}^{X}-a_{j}^{X}\right)}^{2}+{\left(p_{t}^{Y}-a_{j}^{Y}\right)}^{2}+{\left(k_{t}-a_{j}^{Z}\right)}^{2}}}\\ \frac{\partial d_{t}^{j}\left(x_{t},k_{t}\right)}{\partial p_{t}^{Y}}=\frac{p_{t}^{Y}-a_{j}^{Y}}{\sqrt{{\left(p_{t}^{X}-a_{j}^{X}\right)}^{2}+{\left(p_{t}^{Y}-a_{j}^{Y}\right)}^{2}+{\left(k_{t}-a_{j}^{Z}\right)}^{2}}} \end{array}\right.$$

Then, $K={\left[\nabla_{k_{t}}d\left(x_{t},k_{t}\right)\right]}_{1\times N}$ is expressed as:

(33) $$K=\left[\begin{array}{ccccc} \frac{\partial d_{t}^{1}\left(x_{t},k_{t}\right)}{\partial k_{t}} & \ldots & \frac{\partial d_{t}^{j}\left(x_{t},k_{t}\right)}{\partial k_{t}} & \ldots & \frac{\partial d_{t}^{N}\left(x_{t},k_{t}\right)}{\partial k_{t}} \end{array}\right]$$

where:

(34) $$\frac{\partial d_{t}^{j}\left(x_{t},k_{t}\right)}{\partial k_{t}}=\frac{k_{t}-a_{j}^{Z}}{\sqrt{{\left(p_{t}^{X}-a_{j}^{X}\right)}^{2}+{\left(p_{t}^{Y}-a_{j}^{Y}\right)}^{2}+{\left(k_{t}-a_{j}^{Z}\right)}^{2}}}$$

Since $x_{t}$ and $k_{t}$ are within the same function $d(x_{t},k_{t})$, $H_{t}$ and K are both derived from $d(x_{t},k)$. In order to analyze the impacts of $x_{t}$ and $k_{t}$, we decompose the matrix and obtain (31) and (33). For a 3D positioning case, we can combine $H_{t}$ and K together to indicate the 3D position state. However, it is more convenient to separate $H_{t}$ and K to analyze the estimation performance of $x_{t}$ in a 2D positioning case.

If any prior information is unknown to the system, substitute (31) and (33) into (27), then we obtain:

(35) $$J\left(x_{t}\right)=H_{t}\Lambda H_{t}^{T}-H_{t}\Lambda K^{T}\left(K\Lambda K^{T}\right)K\Lambda H_{t}^{T}$$

For the prior information of $k_{t}$, we assume that the target is always above the ground, which is $k_{t}\geq 0$. Thus, we apply the Gamma distribution to indicate the potential distribution of $k_{t}$, where $k_{t}∼G\left(\alpha_{k},\beta_{k}\right)\left(k_{t}\right)=\frac{{\left(\beta_{k}\right)}^{\alpha_{k}}}{\Gamma (\alpha_{k})}{k_{t}}^{\alpha_{k}-1}\exp\left(-\beta_{k}k_{t}\right)$, $\alpha_{k}$ is the shape parameter and $\beta_{k}$ is the rate parameter. For the Gamma distribution, $J_{k}$ is complicated. To obtain an analytical expression, we assume $\alpha_{k}>2$ for simplicity. Then, the Gamma function is $\Gamma \left(\alpha_{k}\right)=\int_{0}^{+\infty }\exp\left(-x\right)x^{\alpha_{k}-1}dx$. Thus, $J_{k}=E{\left(\frac{\partial }{\partial k}G\left(\alpha_{k},\beta_{k}\right)\left(k\right)\right)}^{2}$ is derived as:

(36) $$\begin{array}{cc} J_{k}= & \beta_{k}^{2}-\frac{2\left(\alpha_{k}-1\right)\beta_{k}}{\Gamma (\alpha_{k})}\int_{0}^{+\infty }\beta_{k}^{\alpha_{k}}{k_{t}}^{\alpha_{k}-2}\exp\left(-\beta_{k}k_{t}\right)dk_{t}\\ & +\frac{{\left(\alpha_{k}-1\right)}^{2}}{\Gamma (\alpha_{k})}\int_{0}^{+\infty }\beta_{k}^{\alpha_{k}}{k_{t}}^{\alpha_{k}-3}\exp\left(-\beta_{k}k_{t}\right)dk_{t} \end{array}$$

Use the property $\Gamma \left(\alpha_{k}\right)=\alpha_{k}\Gamma \left(\alpha_{k}-1\right)$, and substitute it into (36); we obtain:

(37) $$J_{k}=\frac{\beta_{k}^{2}}{\alpha_{k}-2}$$

### 4.2. NLOS Impact

We use the vector l to represent the NLOS indicator. In the TOA system, the NLOS delays the wireless packet propagation linearly. Thus, l is the linear auxiliary vector. We assume there are $N_{l}\leq N$ NLOS measurements, and the drift for each measurement is independent of the others, then $L={\left[\nabla_{l}h\right]}_{N_{l}\times N}$ is formulated as:

(38) $$\begin{array}{cc} L & =\left(\begin{array}{ccccc} \frac{\partial h_{t}^{1}}{\partial l_{1}} & \ldots & \frac{\partial h_{t}^{N_{l}}}{\partial l_{1}} & \ldots & \frac{\partial h_{t}^{N}}{\partial l_{1}}\\ \vdots & \ddots & \vdots & \ddots & \vdots \\ \frac{\partial h_{t}^{1}}{\partial l_{N_{l}}} & \ldots & \frac{\partial h_{t}^{N_{l}}}{\partial l_{N_{l}}} & \ldots & \frac{\partial h_{t}^{N}}{\partial l_{N_{l}}} \end{array}\right)\\ & =\left(\begin{array}{cc} I_{N_{l}} & 0 \end{array}\right) \end{array}$$

where $I_{N_{l}}$ is the identity matrix of order $N_{l}$, and the rest is a $N_{l}\times \left(N-N_{l}\right)$ zero matrix due to the independent condition for the LOS measurement. As mentioned before, the zero matrix is mitigated during the calculation, then $L=I_{N_{l}}$. Note that L indicates the sub-matrix of the FIM, which means that the NLOS condition is unknown to the system. Then, the system should also estimate the NLOS parameters together with the position state in the real environment. In this case, the estimated error is propagated and degrades the position estimation.

Since the range drift for the NLOS is also nonnegative, we still use the Gamma distribution as the prior information $l_{m}∼G\left(a_{m},b_{m}\right)\left(l_{m}\right)=\frac{{\left(b_{m}\right)}^{a_{m}}}{\Gamma (a_{m})}l_{m}^{a_{m}-1}\exp\left(-b_{m}l_{m}\right)$ according to [17], where $a_{m}\geq 2$ is the shape parameter, $b_{m}$ is the rate parameter and *m* is the *m*-th NLOS measurement. Similar to (37), we obtain $J_{L}$:

(39) $$J_{L}=\mathrm{diag}\left(\frac{b_{1}^{2}}{a_{1}-2},\ldots ,\frac{b_{m}^{2}}{a_{m}-2},\ldots ,\frac{b_{N_{l}}^{2}}{a_{N_{l}}-2}\right)$$

where $J_{L}$ is the prior matrix for L, which illustrates that the system can use the prior information to determine whether a measurement is LOS or NLOS if this information is unknown. In this case, the estimation error can be reduced to some extent.

## 5. Simulation

We set up several WSN localization simulations to evaluate the analytical performance. In each simulation, we consider several different factors, e.g., the recursive process during the target tracking, estimations with and without considering l and $k_{t}$. To make the results clear, we mark the CRLBs for different situations by adding superscripts and subscripts, which can be depicted as ${\mathrm{CRLB}}_{\ldots }^{\ldots }$. The subscripts indicate the considered vectors, including the state vector and the auxiliary vector. This means that the system contains other factors in the ranging measurement, e.g., relative height or NLOS measurement or both. The superscripts indicate the available prior information of the related vectors. For instance, if we want to simulate the estimation with the NLOS range drift, the results of the CRLB are marked by ${\mathrm{CRLB}}_{x_{t},l}$. Additionally, if the prior information of $x_{t}$ is attained in the simulation, the results are marked by ${\mathrm{CRLB}}_{x_{t},l}^{x_{t}}$. For the recursive estimation, we use the notation ${\mathrm{CRLB}}_{x_{t},l}^{x_{t-1},l}$ to indicate the results, which considers the prior state $x_{t-1}$. Note that, the superscript of $x_{t}$ in ${\mathrm{CRLB}}_{\ldots }^{x_{t},\ldots }$ indicates that we have arbitrary prior knowledge of $x_{t}$. This information is provided directly by the system instead of recursive estimation; while the superscript of $x_{t-1}$ in ${\mathrm{CRLB}}_{\ldots }^{x_{t-1},\ldots }$ also means the prior knowledge of $x_{t}$. However, such information is obtained based on the previous estimation of $x_{t-1}$ in the recursive target tracking. Thus, we use ${\mathrm{CRLB}}_{\ldots }^{x_{t},\ldots }$ to analyze the static positioning scenarios and use ${\mathrm{CRLB}}_{\ldots }^{x_{t-1},\ldots }$ to analyze the dynamic continuous target tracking scenarios. Some CRLB notations that appear in the following sections are listed below.

- ${\mathrm{CRLB}}_{x_{t}}$: Classical CRLB without any prior information, relative height or NLOS measurement
- ${\mathrm{CRLB}}_{x_{t},k_{t}}$: CRLB when the relative height exists
- ${\mathrm{CRLB}}_{x_{t},l}$: CRLB with the NLOS measurement
- ${\mathrm{CRLB}}_{x_{t},k_{t},l}$: CRLB both contains the relative height and NLOS measurement
- ${\mathrm{CRLB}}_{x_{t},k_{t}}^{k_{t}}$: CRLB with the prior information of the relative height
- ${\mathrm{CRLB}}_{x_{t},l}^{l}$: CRLB with the prior information of NLOS measurement
- ${\mathrm{CRLB}}_{x_{t},\ldots }^{x_{t},\ldots }$: CRLB with the arbitrary prior information of $x_{t}$ in the static scenario
- ${\mathrm{CRLB}}_{x_{t},\ldots }^{x_{t-1},\ldots }$: CRLB with the prior information based on $x_{t-1}$ in the dynamic scenario

To approach the real environment, we initially set the related parameters according to the test building in [27]. Since the localization system can be affected by many factors, we tune the parameters to provide a comprehensive analysis in the following simulations, e.g., $k_{t}$, l and the number of anchors. To better understand the performances in multiple environments, we uniformly locate the anchors in the SPED evaluation. Additionally, we evaluate the performances considering the relative height, NLOS and recursive estimation based on randomly-deployed anchors and Monte Carlo simulation to draw a general performance. In the real experiment, the anchors are randomly deployed throughout the whole building.

### 5.1. Spatial Position Error Distribution

In the first simulation, a $100\times 100\;m^{2}$ playing field with four anchors is constructed. All of the anchors are deployed on the roof being 2.5 m high, and the target is about 0.5∼1 m. To approach the real applications, we use several statistical results according to [27]. We set the relative height as the constant value 1.5 m. The range error for each anchor follows zero-mean Gaussian distribution $v_{t}^{j}∼N\left(0,R_{t}^{j}\right)$, where $R_{t}^{j}$ is the variance of $v_{t}^{j}$ and is set to $5^{2}$. The range drift for the NLOS measurements is set to 2 m. For the prior information, $k_{t}∼G\left(2.5,2\right)\left(k\right)$. The prior information of the NLOS range drift $l_{m}$ is $l_{m}∼G\left(3.5,1.8\right)\left(l_{m}\right)$. For the position state prior information, we assume that the prediction function is a linear static identity matrix with the zero-mean Gaussian prediction noise $q_{t}∼N\left(0,Q_{t}\right)$, where $Q_{t}=\mathrm{diag}\left(\sigma_{x}^{2},\sigma_{y}^{2}\right)$ is the covariance of $q_{t}$. We assume $\sigma_{x}=\sigma_{y}=2$ m.

**Figure 2:**
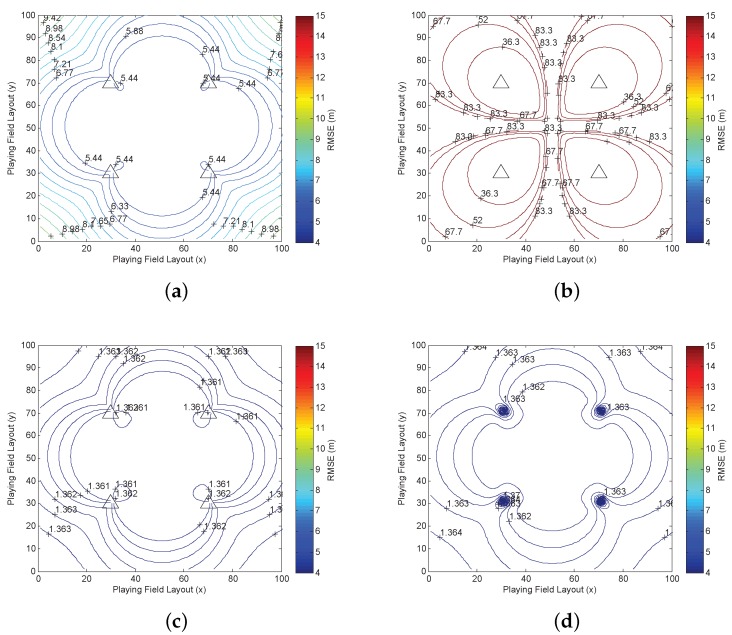
The spatial position error distribution (SPED) results of the CRLB with and without prior information. (**a**) The SPED of ${\mathrm{CRLB}}_{x_{t}}$; (**b**) the SPED of ${\mathrm{CRLB}}_{x_{t},k_{t},l}$; (**c**) the SPED of ${\mathrm{CRLB}}_{x_{t}}^{x_{t}}$; (**d**) the SPED of ${\mathrm{CRLB}}_{x_{t},k_{t},l}^{x_{t},k_{t},l}$.

We apply the CRLB to indicate the optimal squared error, which is $\sqrt{\mathrm{tr}(J^{-1}\left(x_{t}\right))}$. To illustrate the target-anchor geometry effect for the 2D localization system in the playing field, we employ $\sqrt{\mathrm{tr}(J^{-1}\left(x_{t}\right))}$ to depict the spatial position error distribution (SPED) [28]. The SPED is defined as the distribution of the position error for every possible target position, which estimates the positions point by point in the whole playing field and draws statistical results. Thus, every position in the playing field is assumed as the target position during the simulation. The SPED is derived according to all of the statistical results of the estimation error of all of the positions in the playing field. It illustrates how the performance changes according to the target-anchor (RX-TX) geometry effect. When the anchor positions and the error model change, the SPED changes accordingly, which helps to understand the relationship between the anchor deployment and the algorithm. The SPED results are represented by the contours in Figure 2, where the four anchor positions are marked by triangles.

In Figure 2a, the SPED is drawn based on the classical CRLB (${\mathrm{CRLB}}_{x_{t}}$) in which the unknown vector is only $x_{t}$, and no relative height or NLOS propagation is introduced. When both $k_{t}$ and l are introduced in the simulation, the dimension of the estimated vector is increased again. Then, a special pattern of the contours is drawn in Figure 2b, which demonstrates that the estimation error is propagated and increased if more unknown factors are introduced. Each anchor seems to be a center of an independent contour area, and the estimation error is extremely larger than Figure 2a, which indicates the strong uncertainty and a special geometric relationship between the target position and the anchor positions. Since more factors cannot be ignored in the complicated environment, the localization problem turns out to be a high dimensional estimation problem. It is quite possible that the squared error of the high dimensional estimation is larger than the low dimensional estimation, as the probability of the wrong estimation increases if more unknown parameters appear. Additionally, it also imposes new error on the original $x_{t}$ estimation error. However, such a high dimensional localization problem cannot happen in the real world. On the one hand, the calculation complexity is increased dramatically in the real system, and only $x_{t}$ is useful, which is a waste of computational resources. On the other hand, the algorithm designers ignore some unimportant factors, simplify the calculation and increase the estimation accuracy based on the prior knowledge.

Then, the prior information of $x_{t}$, $k_{t}$ and l is used in the CRLB to improve the estimation accuracy. The SPEDs with prior information are depicted in Figure 2c,d. When the prior state information of $x_{t}$ is introduced in Figure 2c, the estimation error is reduced effectively from more than 5 m in Figure 2a to 1.4 m below. In addition, the position error is almost the same everywhere, although the geometric pattern is similar to Figure 2a, which indicates that the geometric impact is reduced by using the prior information of $x_{t}$. In Figure 2d, we assume that all of the prior information is available, then the estimation error is reduced to below 1.4 m, just as Figure 2c. Although Figure 2d still has a geometric pattern, which is similar to Figure 2c, the errors in different positions are almost the same. Thus, the geometric effect is actually reduced. In addition, since the estimation error is not further reduced, the results in Figure 2d can be the limits based on the prior information. It is also demonstrated that the prior information of $x_{t}$ significantly reduces the estimation error and is the most important prior information especially in the recursive estimation. For the other two parts of the prior information, they are used for the improvement of the impacts of the relative height and the NLOS effects.

### 5.2. Impact of Relative Height

**Figure 3:**
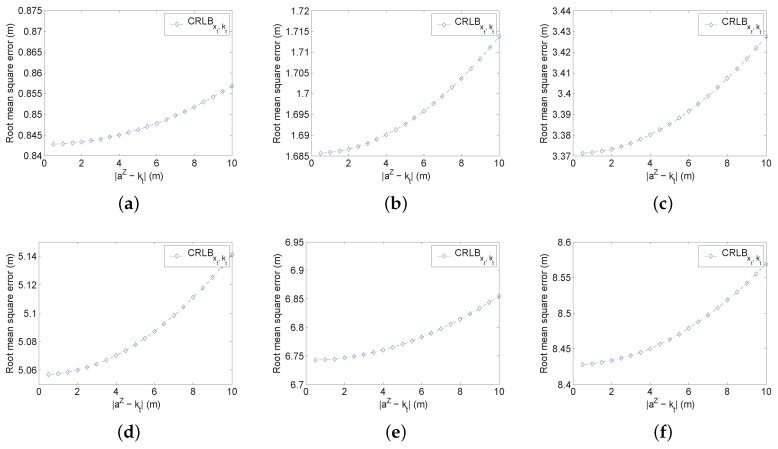
The evaluation of the impact of $k_{t}$. (**a**) $v_{t}^{j}∼N\left(0,0.5^{2}\right)$; (**b**) $v_{t}^{j}∼N\left(0,1^{2}\right)$; (**c**) $v_{t}^{j}∼N\left(0,2^{2}\right)$; (**d**) $v_{t}^{j}∼N\left(0,3^{2}\right)$; (**e**) $v_{t}^{j}∼N\left(0,4^{2}\right)$; (**f**) $v_{t}^{j}∼N\left(0,5^{2}\right)$.

In this simulation, we evaluate the impact of the relative height independently. For the indoor localization, the relative height cannot be too high unless the room is tall enough. Thus, we choose the value of $k_{t}$ between 0.5 m and 10 m. The simulation evaluates ${\mathrm{CRLB}}_{x_{t},k_{t}}$ and also ${\mathrm{CRLB}}_{x_{t}}$ as a comparison. To illustrate the general relationship between the relative height and the measurement noise, we also tune the variance of the noise from $0.5^{2}$ to $5^{2}$ and $k_{t}$ from 0.5 m and 10 m. In this simulation, we run 1000 Monte Carlo experiments. In each experiment, we choose a random position for analysis. The averaged results are listed in Figure 3.

It is clearly observed that the estimation error rises according to the increase of $k_{t}$. However, the increased value is quite small. Take Figure 3c for instance; the increased error is only 0.06 m when $k_{t}$ is tuned from 0.5 m to 10 m, and the estimation error is 3.43 m by that time. Therefore, the value of $k_{t}$ does not affect the estimation accuracy too much. On the other hand, the existence of $k_{t}$ does affect the performance no matter what the value of ${\mathrm{CRLB}}_{x_{t}}$ is. Compare the results in Figure 3c; $k_{t}$ affects the estimation with a typical certain value. When $k_{t}$ is small, the average RMSE of ${\mathrm{CRLB}}_{x_{t}}$ is 1.4390 m, but the average RMSE of ${\mathrm{CRLB}}_{x_{t},k_{t}=0.5}$ is 1.6856 m and ${\mathrm{CRLB}}_{x_{t},k_{t}=10}$ is 1.7137 m. According to the analytical results, when the relative height is introduced into the simulation, there is a gap between 2D localization without the relative height. Additionally, the gap becomes larger with the increased value of the measurement noise. Take $v_{t}^{j}∼N\left(0,0.5^{2}\right)$ for instance; when $k_{t}=0$, the average RMSE is only 0.84 m. When $v_{t}^{j}∼N\left(0,5^{2}\right)$, the average RMSE without the relative height is about 8.3 m, which is almost 10-times that of the low noise. Thus, the relative height really affects the estimation no matter the value.

**Figure 4:**
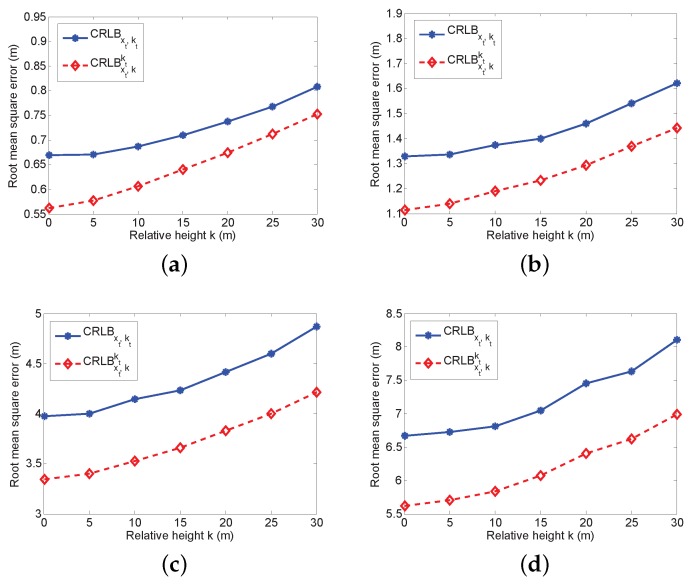
The evaluation of the impact of $k_{t}$ with the multiple noise environment and prior knowledge of *k*. (**a**) $v_{t}^{j}∼N\left(0,0.5^{2}\right)$; (**b**) $v_{t}^{j}∼N\left(0,1^{2}\right)$; (**c**) $v_{t}^{j}∼N\left(0,3^{2}\right)$; (**d**) $v_{t}^{j}∼N\left(0,5^{2}\right)$.

In addition, we evaluate the impact of the prior knowledge of $k_{t}$ in the multiple noise environment. The simulation results are presented in Figure 4. It is clearly observed that the prior knowledge can significantly improve the RMSE. If $v_{t}^{j}∼N\left(0,5^{2}\right)$, the prior information of $k_{t}$ can even reduce 1 m RMSE. Additionally, if the error is small, the improvement is only a little bit. However, with the increased value of the relative height, the RMSE rises accordingly even with the prior information. For the real application, the relative height can be within 10 m, in which the improvement can effectively reduce the RMSE to a reasonable range. Therefore, even if the impact of the relative height is limited, it is still necessary to employ the prior information.

### 5.3. Impact of NLOS

**Figure 5:**
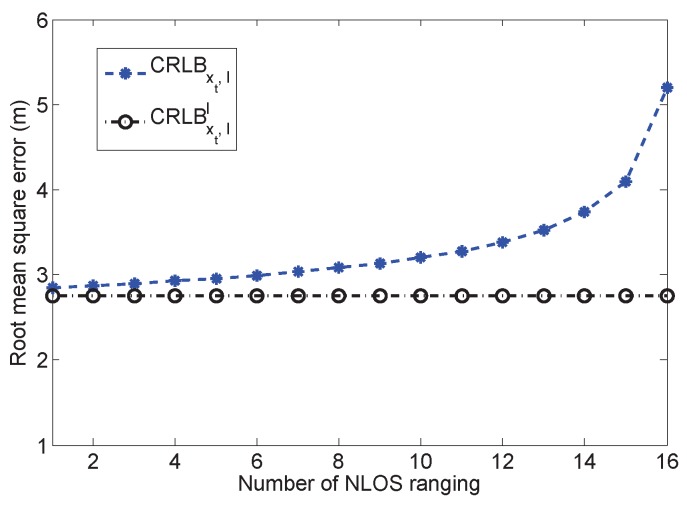
The impact of the number of *l*.

The analysis of NLOS measurements has been mentioned in several literature works [17,18]. Here, we evaluate the impact of the number of NLOS measurements through simulations. The playing field is still $100\times 100\;m^{2}$ with 16 randomly-deployed anchors. We set up 1000 Monte Carlo runs, and a random set of anchors is chosen to have NLOS measurements. We tune the number of NLOS measurements from 1 to 16 and evaluate the performance of ${\mathrm{CRLB}}_{x_{t}}$ and ${\mathrm{CRLB}}_{x_{t},l}^{l}$. The averaged results are depicted in Figure 5. It is observed that the RMSE of ${\mathrm{CRLB}}_{x_{t},l}$ increases according to the rise of the NLOS measurement number. It is also possible to have useless estimation when all of the measurements are NLOS, just as mentioned in [17]. When there is only one NLOS measurement, the estimation error reaches 3 m. Additionally, the error is increased to 5 m if the number of NLOS measurements is changed to 16. In real applications, the number of NLOS measurements is unknown, and the system has to calculate all of the parameters from all of the measurements. Thus, the estimation error is quite high without the help of prior information. In addition, with the prior information of l, the estimation error is almost a constant value. Thus, the prior information of the NLOS measurement can successfully reduce the NLOS effect, which is applicable for real applications. Such prior information can be a combination of the building layout information and the wireless propagation model, e.g., map-matching-based algorithms [13]. Additionally, several literature works have proposed many algorithms that either use pre-assumptions or estimate the parameters to obtain the NLOS information. The NLOS-related methods are beyond the scope of this paper; please refer to other research works in [20,23,29].

### 5.4. Map Assist Localization System

**Figure 6:**
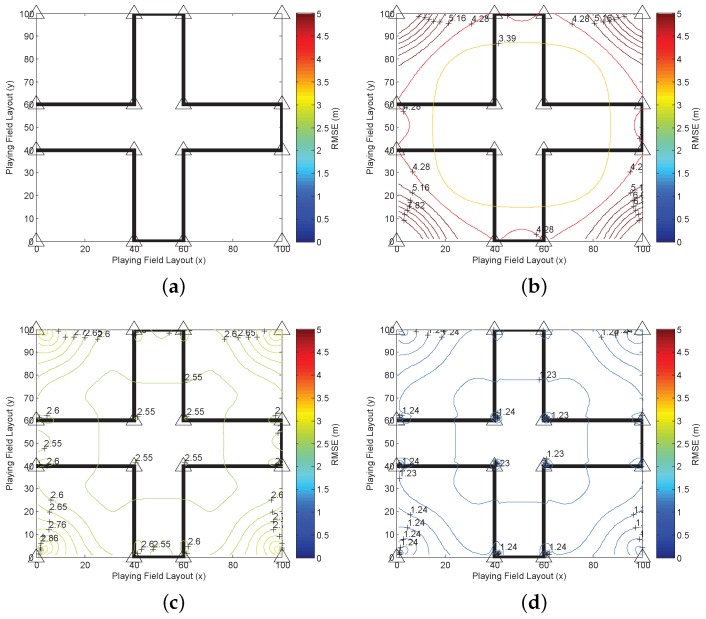
The simulation of a building layout. (**a**) Anchor deployment of the playing field; (**b**) the SPED of ${\mathrm{CRLB}}_{x_{t},k_{t},l}$; (**c**) the SPED of ${\mathrm{CRLB}}_{x_{t},k_{t},l}^{k_{t},l}$; (**d**) the SPED of ${\mathrm{CRLB}}_{x_{t},k_{t},l}^{x_{t},k_{t},l}$.

In the following simulation, we evaluate a practical scenario where the layout of the building is involved. The playing field is still $100\times 100\;m^{2}$. There are four big rooms located at four corners of the playing field. The area for each room is $40\times 40\;m^{2}$. The rest of the playing field is the hallways. Four anchors are deployed inside each room and placed at the four corners. Thus, 16 anchors are uniformly deployed in the playing field, which is depicted in Figure 6a. The triangles mark the positions of anchors. The LOS measurements can only be obtained in one room with four associate anchors, and others measurements are NLOS. For the positions in the hallways, all of the measurements are NLOS rangings.

All of the anchors are deployed on the roof with relative height 1∼2 m to the target. The distributions of the range error and the related prior information are the same in Figure 2. The range drift for the NLOS measurements depends on the signal transmission from the anchor to the target. According to our previous research, the average positive NLOS drift of the signal through one wall is set to 2 m and to 5m for the signal through two or three walls if the signal can be detected [30]. The prior information of the NLOS range drift $l_{m}$ depends on the signal transmission. Here, we use the conclusions based on the experimental models in [27]: the distribution for the signal transmission through one wall is $l_{m}∼G\left(3.5,1.8\right)\left(l_{m}\right)$; the distribution for the signal transmission through more than two walls is $l_{m}∼G\left(4.5,2.2\right)\left(l_{m}\right)$. In this simulation, we evaluate the SPED of ${\mathrm{CRLB}}_{x_{t},k_{t},l}$, ${\mathrm{CRLB}}_{x_{t},k_{t},l}^{k_{t},l}$ and ${\mathrm{CRLB}}_{x_{t},k_{t},l}^{x_{t},k_{t},l}$, which means that every position is calculated, and the error contours are drawn to indicate the target-anchor geometry effect. The results are depicted in Figure 6.

For numerical comparison, the RMSE in Figure 6b is higher than in Figure 6c,d, which is more than 3.39 m in the central area. Additionally, the error becomes higher and higher when the position is approaching the corner, which is more than 8 m. Due to the lack of prior information, the geometric shape does not have special characteristics, which are related to rooms or corridors. The contours are almost like rectangles located in the center of the playing field. When the prior information of $k_{t}$ and l is introduced, the accuracy is significantly improved, which is reduced to 2.55 m on average. The geometric shapes of the contours are different in the rooms and hallways. This indicates that the localization algorithms using the prior knowledge of NLOS conditions based on the building layout information and the NLOS identification and mitigation methods can reasonably improve the estimation performance. Thus, the layout information in the building map is an important information source for localizations. When the prior information of $x_{t}$ is introduced in the estimation as indicated in Figure 6d, the RMSE is further reduced, which is 1.235 m in almost all of the playing field where the target-anchor geometry effect is reduced effectively.

### 5.5. Bayesian-Based Target Tracking Estimation

In this simulation, we evaluate the performance of the recursive Bayesian estimation for target tracking. Since the impacts of $k_{t}$ and l are extensively analyzed above, this section mainly focuses on analyzing the impact of $x_{t-1}$. The playing field is still $100\times 100\;m^{2}$, and 16 anchors are deployed randomly in the field. We evaluate the CRLB in three scenarios: The first one considers NLOS and relative height measurement; the second scenario considers only the relative height; the third scenario only has NLOS measurement. In each scenario, both CRLB without prior information and the recursive CRLB with prior information based on (27) are analyzed. Therefore, we evaluate ${\mathrm{CRLB}}_{x_{t},k_{t},l}$ and ${\mathrm{CRLB}}_{x_{t},k_{t},l}^{x_{t-1},k_{t},l}$ in the first scenario, ${\mathrm{CRLB}}_{x_{t},k_{t}}$ and ${\mathrm{CRLB}}_{x_{t},k_{t}}^{x_{t-1},k_{t}}$ in the second scenario and ${\mathrm{CRLB}}_{x_{t},l}$ and ${\mathrm{CRLB}}_{x_{t},l}^{x_{t-1},l}$ in the third scenario. We assume that the prior information is unknown initially in the recursive estimation. This information can only be obtained recursively after the initial estimation is obtained. Then, the recursive estimation is applied.

**Figure 7:**
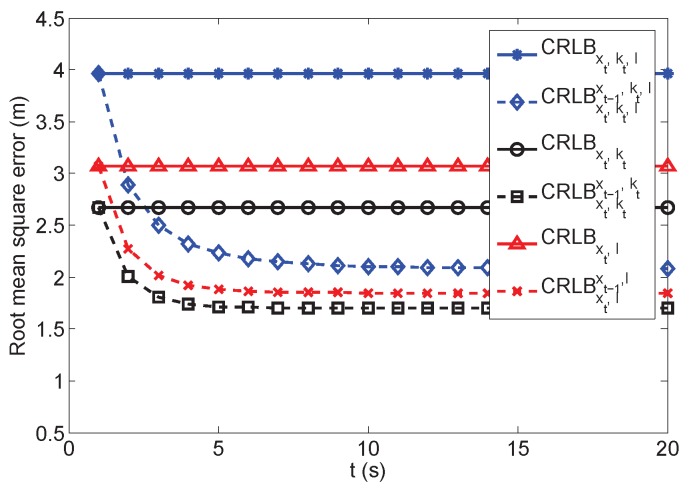
Sequential estimation lower bound.

We run 1000 Monte Carlo simulations, and the target moves in a separate random path in each simulation. In addition, the target can also be static. Since $x_{t-1}$ can also be estimated in the static scenario and be used for recursive estimation, the analysis results are the same as the dynamic target tracking scenarios. The estimation results are averaged and represented by the RMSE in Figure 7. There are three solid parallel straight lines, which indicate the estimations without prior information: ${\mathrm{CRLB}}_{x_{t},k_{t},l}$, ${\mathrm{CRLB}}_{x_{t},k_{t}}$ and ${\mathrm{CRLB}}_{x_{t},l}$. The three other dashed curves illustrate the recursive estimations according to time steps, which are ${\mathrm{CRLB}}_{x_{t},k_{t},l}^{x_{t-1},k_{t},l}$, ${\mathrm{CRLB}}_{x_{t},k_{t}}^{x_{t-1},k_{t}}$ and ${\mathrm{CRLB}}_{x_{t},l}^{x_{t-1},l}$.

The Bayesian recursive estimation method with related prior information effectively reduced the estimation as indicated in Figure 7. The RMSEs of the three curves gradually converge to low values according to time steps. The impacts of the relative heights and the NLOS drifts still degrade the estimation performance. Even with the recursive estimation, the estimation error cannot be further reduced, where the ${\mathrm{CRLB}}_{x_{t},k_{t},l}^{x_{t-1},k_{t},l}$ is 0.5 m larger than ${\mathrm{CRLB}}_{x_{t},k_{t}}^{x_{t-1},k_{t}}$ when t=20.

### 5.6. Multiple Anchors

**Figure 8:**
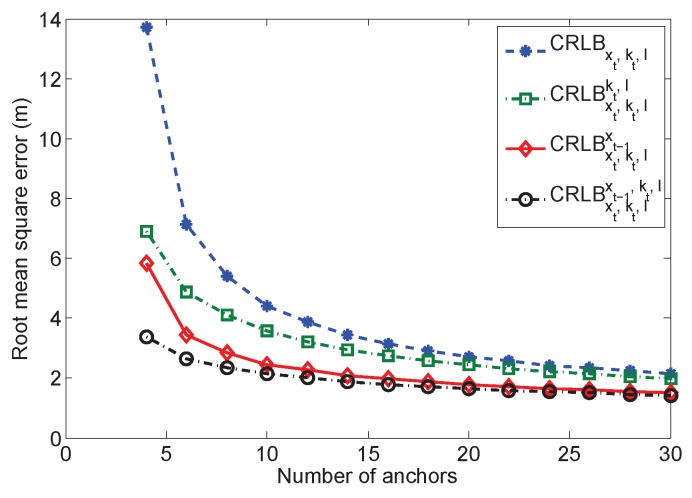
Cramér–Rao lower bound with different number of anchors.

The relationship between the CRLB and the number of anchors is illustrated in Figure 8. We randomly deploy multiple anchors in the playing field. The number of anchors is adapted from four to 30. Both $k_{t}$ and l are considered in this simulation. We assume that all of the measurements are NLOS. In this simulation, we evaluate ${\mathrm{CRLB}}_{x_{t},k_{t},l}$, ${\mathrm{CRLB}}_{x_{t},k_{t},l}^{k_{t},l}$, ${\mathrm{CRLB}}_{x_{t},k_{t},l}^{x_{t-1}}$ and ${\mathrm{CRLB}}_{x_{t},k_{t},l}^{x_{t-1},k_{t},l}$. With a few anchors, ${\mathrm{CRLB}}_{x_{t},k_{t},l}$ contains large error, which achieves more than 15 m. When the number of anchors is four, the prior information of $k_{t}$ and l can reduce much of the error as indicated by the curve of ${\mathrm{CRLB}}_{x_{t},k_{t},l}^{k_{t},l}$. Furthermore, the recursive estimation based on ${\mathrm{CRLB}}_{x_{t},k_{t},l}^{x_{t-1}}$ can reduce much of the error, which is even smaller than ${\mathrm{CRLB}}_{x_{t},k_{t},l}^{k_{t},l}$. The error of the recursive estimation based on ${\mathrm{CRLB}}_{x_{t},k_{t},l}^{x_{t-1},k_{t},l}$ is only a little lower than ${\mathrm{CRLB}}_{x_{t},k_{t},l}^{x_{t-1}}$. It is demonstrated that compared to the prior information of $k_{t}$ and l, $x_{t-1}$ is the dominant factor for reducing the estimation error. However, when the number of anchors is increased, all of the curves converge gradually to a low value. In this case, the prior information does not improve much for the estimation. Thus, even if the prior information is not available, using more observations still improves the estimation accuracy effectively.

### 5.7. Practical Evaluation

**Figure 9:**
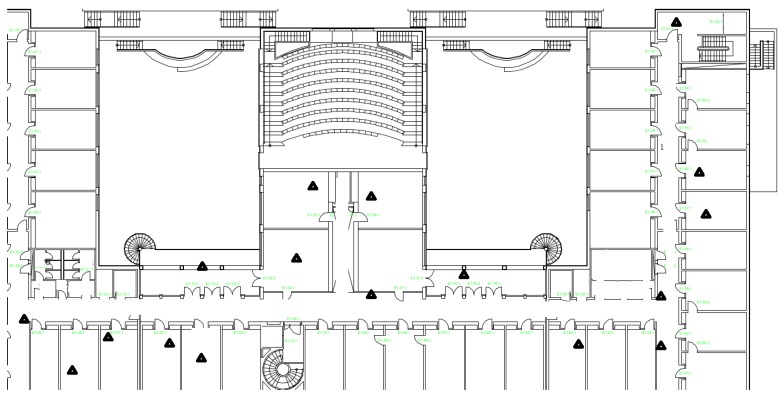
Building layout for the indoor localization experiment and the robot trajectory. The triangles mark the positions of sensor nodes, which are placed either in the offices or along the corridor.

For practical usage, we employ the CRLB to evaluate a reference system. In this system, we deployed 17 wireless sensor nodes either along the corridor or in the offices of the research building. A robot carrying a sensor node as a target moved along the corridor of the building with constant speed while recording its own positions [31]. All sensors are integrated with the nanoPAN 5375 RF (Nanotron Tech. GmbH, Berlin, Germany) module with a 2.4-GHz transceiver and a 1-Mbps data rate for range measurement, the LPC 2387 as the micro-controller (Nanotron Tech. GmbH, Berlin, Germany) and the CC1101 900-MHz transceiver (Nanotron Tech. GmbH, Berlin, Germany) as the radio transceiver for communication. The data collected from sensor nodes are also range measurement values, which are based on TOA. Figure 9 depicts the map of our experimental building. The triangles, which are randomly deployed, mark the sensor node positions.

**Figure 10:**
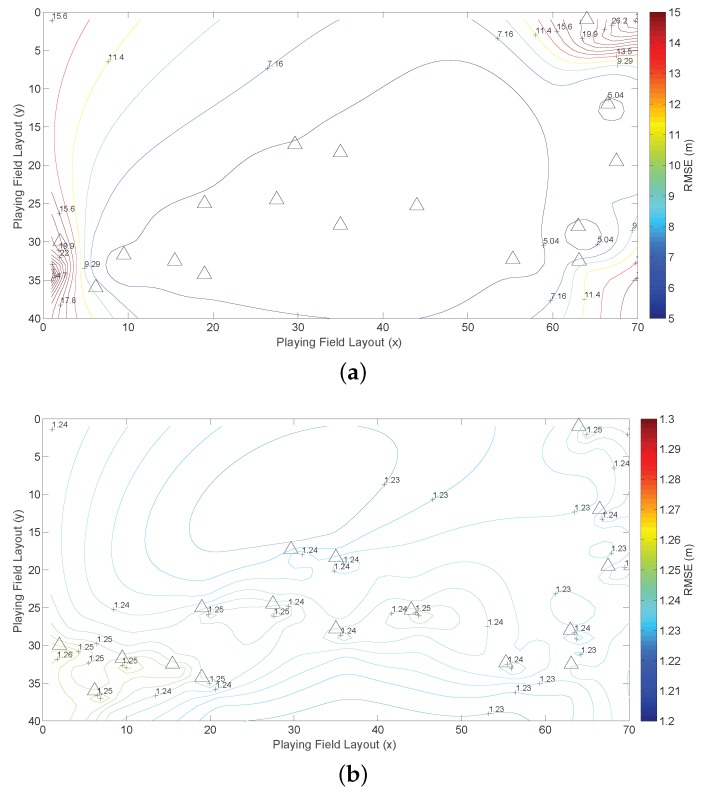
The real system evaluation. (**a**) SPED without prior knowledge; (**b**) SPED with prior knowledge.

According to the collected data and map information, we construct the distribution model for both LOS and NLOS measurements, which are a zero-mean Gaussian distribution $v_{t}^{j}∼N\left(0,R_{t}^{j}\right)$; $R_{t}^{j}$ is set to $5^{2}$ for LOS measurement; and the NLOS measurements drift is set to 2 m. Then, the SPEDs with or without prior knowledge are depicted in Figure 10. As illustrated in Figure 10a, the overall estimation error can be extremely high due to the measurement error. In addition, the anchor-target geometry shape is seriously distorted due to the randomly-deployed anchor. In the corners of the building, the RMSEs are changing rapidly. However, with the help of prior information, we can effectively reduce the error to about 1.25 m.

### 5.8. Discussion

In this section, we use our proposed method to evaluate the optimal performance in multiple environments. It can be observed that our method can adapt the estimated parameters to fit the practical environment. In addition, the scalable architecture can effectively fuse multiple pieces of information, which outperforms other methods, which only consider parts of the information. In Table 2, we compare our method to three mainly used CRLBs, which are generalized CRLB [17], conditional CRLB [16] and equivalent CRLB [18]. The first row in Table 2 indicates the considered information, and the word “prior” means the related prior information. As illustrated in the table, the generalized CRLB can only analyze the NLOS effect. The conditional CRLB provides the formulation of the state vector instead of other factors. The equivalent CRLB exploits more information. However, it is a closed form formulation, which is not scalable and cannot fuse the relative height information.

## 6. Conclusions

In this paper, we propose a scalable analyzing method for a WSN localization system, which can fuse multiple heterogeneous information to indicate the optimal performance. Theoretically, we divide the estimated vector θ into three parts: the estimated state vector and two auxiliary vectors. The recursive formulation of FIM is provided, which fully considers all of the possible factors that may influence the estimation accuracy, and it exploits all of the available information to derive the fundamental limits. It is a suitable tool to indicate the optimal estimation bound of practical systems.

We employ our theoretical contribution to analyze the TOA range-based WSN localization system. The impacts of the height $k_{t}$ and NLOS range drift l are considered as the auxiliary vectors. The target-anchor geometry, prior information and the recursive form are extensively analyzed in the simulation. In addition, we employ a real test-bed and practical data to evaluate the overall performance. In the simulation, we find that both the relative height and NLOS impact can heavily degrade the estimation performance. However, many pieces of available information can improve it, e.g., the prior state distribution, the prior knowledge of the building and multiple anchors.

According to the simulation and experimental demonstration, the proposed CRLB is a general framework for analyzing the WSN localization systems, and it is not restricted to any specific technique. Future work will use the scalable CRLB to exploit other localization techniques and in other complicated environments to find potential factors that may influence the performance.

## Figures and Tables

**Table 1:** Notations and explanations.

$x_{t}$	The target position state	$h_{t}$	The measurement function
$a_{j}$	The target position vector	θ	Unknown state vector
$k_{t}$	The relative height vector	J(θ)	Fisher information matrix
l	The NLOS drift	$J_{P}$	The prior matrix
$z_{t}$	The measurement vector	$J_{D}$	The measurement matrix
$q_{t}$	The prediction noise	$J_{K}$	Nonlinear auxiliary matrix
$v_{t}$	The measurement noise	$J_{L}$	Linear auxiliary matrix
$d^{j}\left(\right)$	The distance function	$J_{S}$	State matrix
$f_{t}\left(\right)$	The prediction function	$J_{A}$	Auxiliary matrix

**Table 2:** Algorithm comparisons.

Algorithm	State	Prior State	NLOS	Prior NLOS	Relative Height	Prior Height
Generalized CRLB			√	√		
Conditional CRLB	√	√				
Equivalent CRLB	√	√	√	√		
Our Method	√	√	√	√	√	√
